# Supplementary figures and images for: Colour change of twig-mimicking peppered moth larvae is a continuous reaction norm that increases camouflage against avian predators
Source: PeerJ. 2017 Nov 14;5:e3999. doi: 10.7717/peerj.3999 (PMC5691783; doi:10.7717/peerj.3999)

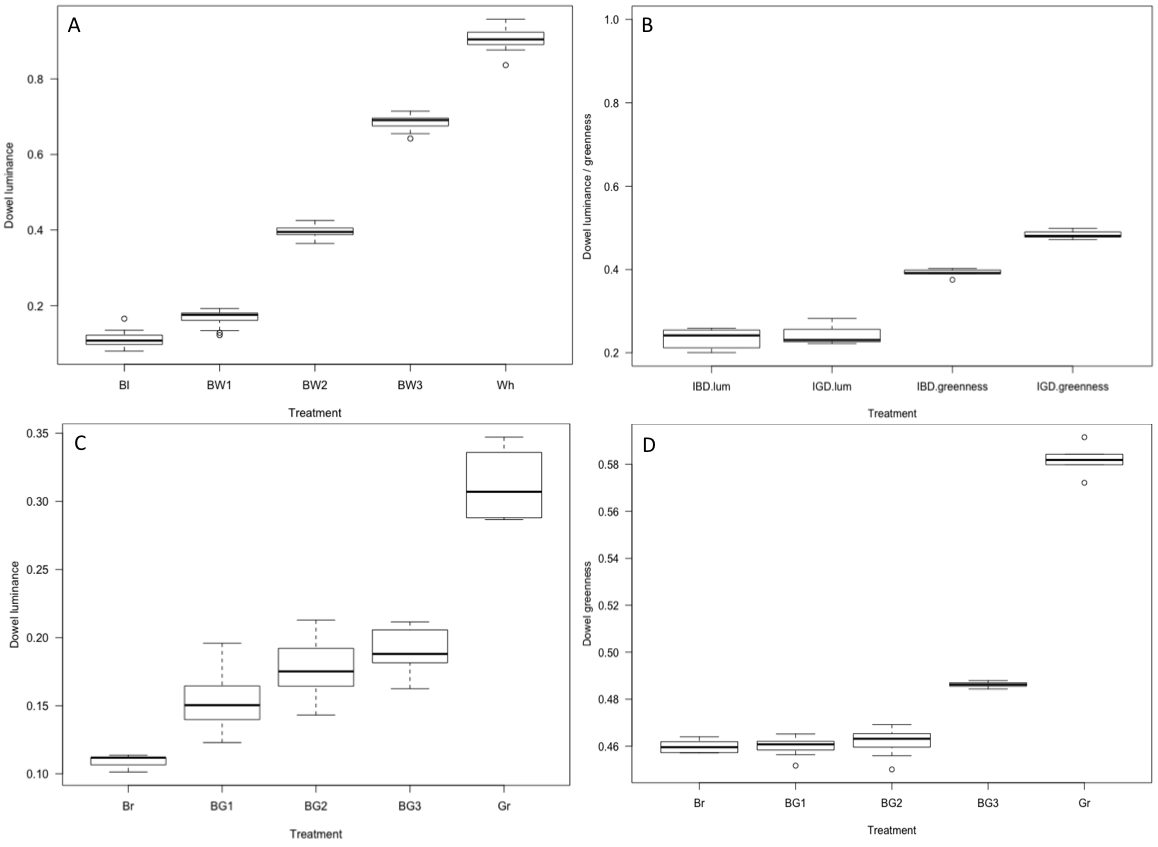

Supplement: Figure S1 — (A) Distribution of dowel luminance and greenness. Luminance of dowels used in luminance gradient experiment; (B) Luminance and greenness of isoluminant brown (IBD) and isoluminant green (IGD) dowels; (C) Luminance of dowels used in colour and luminance gradient experiment; (D) Greenness of dowels used in colour and luminance gradient experiment. For explanation of treatment codes see Table S1. [file peerj-05-3999-s001.png]

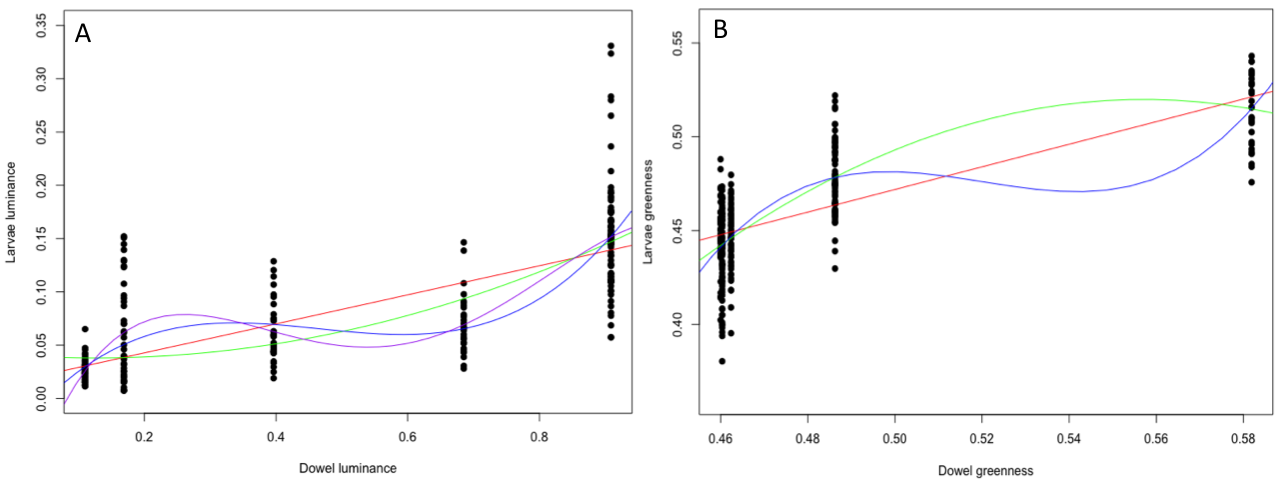

Supplement: Figure S2 — (A) Scatterplot of dowel and larvae luminance from luminance experiment. (B) Scatterplot of dowel and larvae greenness from colour experiment. Polynomial models represented in both panels (A) and (B) by colours: 1st order (red), 2nd order (green), 3rd order (blue), 4th order (purple). [file peerj-05-3999-s002.png]

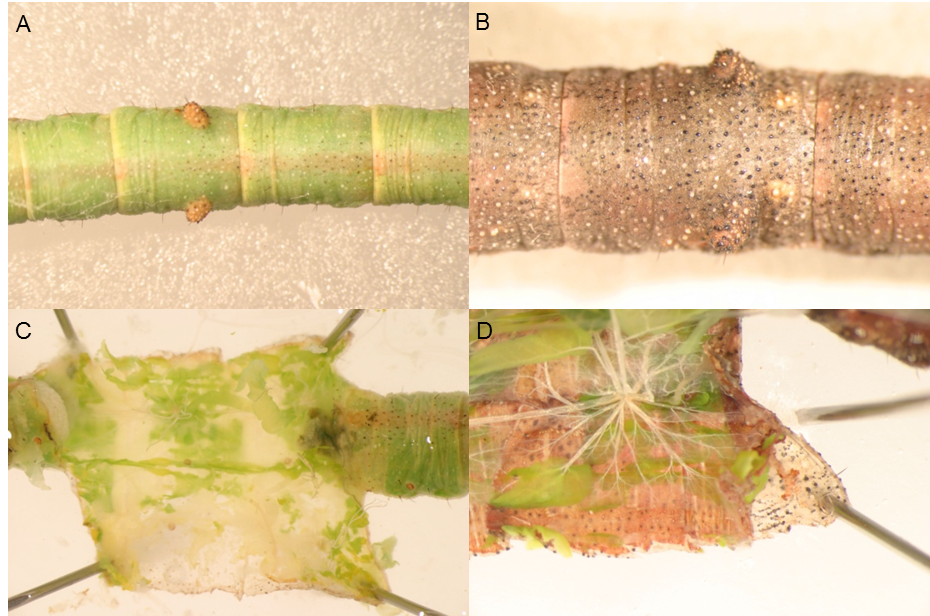

Supplement: Figure S4 — External dorsal surface of green (A) and brown (B) larvae. Dissection of the same larvae reveals that the primary colour in green phenotypes comes from underlying fatty tissue visible through translucent cuticular and epidermal layers (C). In brown phenotypes, there is less green tissue, the epidermis is reddish brown, and the cuticle has pronounced black spots (D). [file peerj-05-3999-s004.png]
