# Supplementary material for: Colour change of twig-mimicking peppered moth larvae is a continuous reaction norm that increases camouflage against avian predators
Source: PeerJ. 2017 Nov 14;5:e3999. doi: 10.7717/peerj.3999 (PMC5691783; doi:10.7717/peerj.3999)
Supplement: Figure S3 — Photographs of the final instar larvae under treatment 0G (0% Green, 100% brown dowel proportions). [file peerj-05-3999-s003.docx]

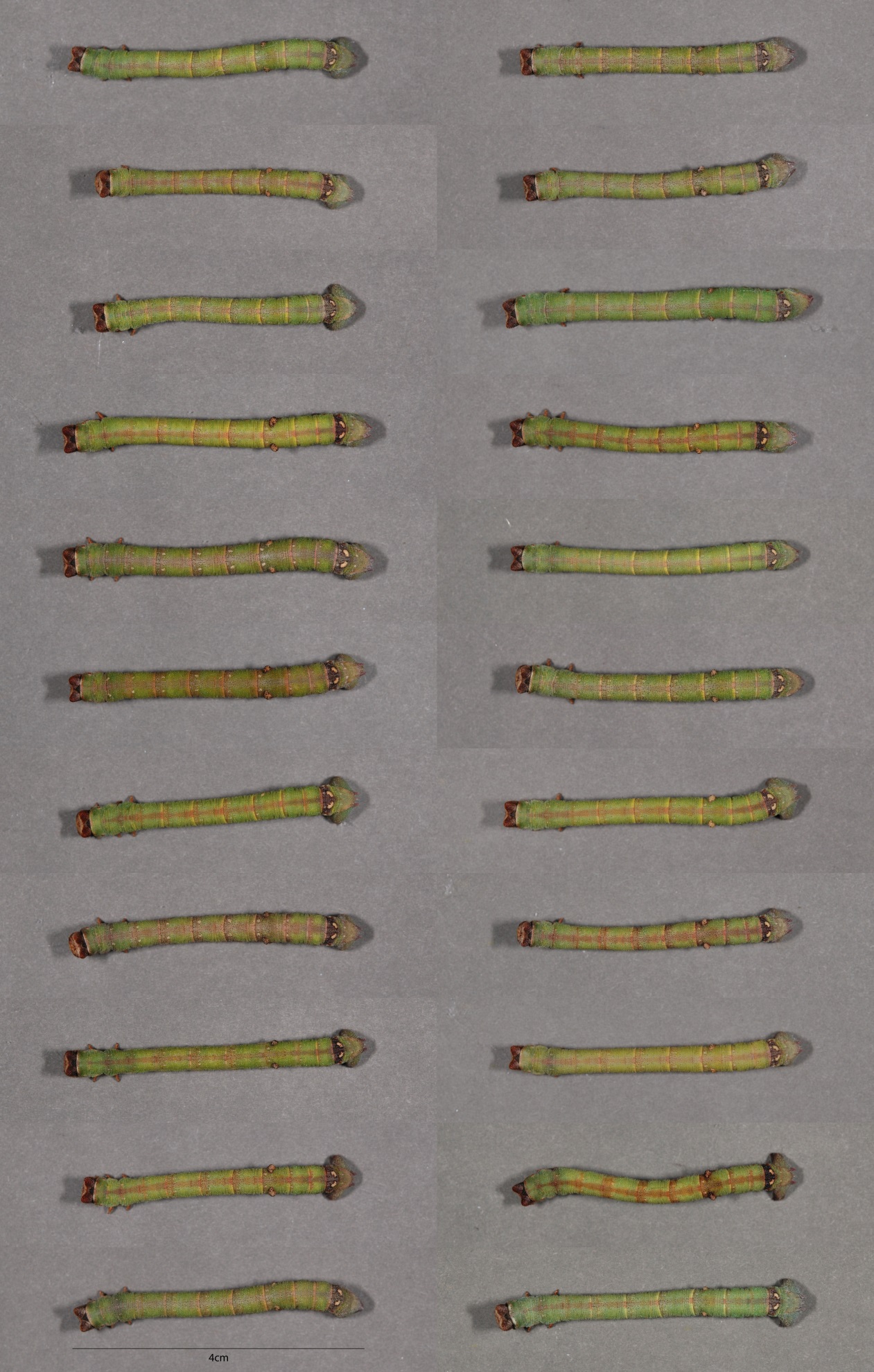


**Fig. S3A. Heterogeneous environment treatment**. Photographs of the final instar larvae under treatment 100G (100% Green, 0% brown dowel proportions).


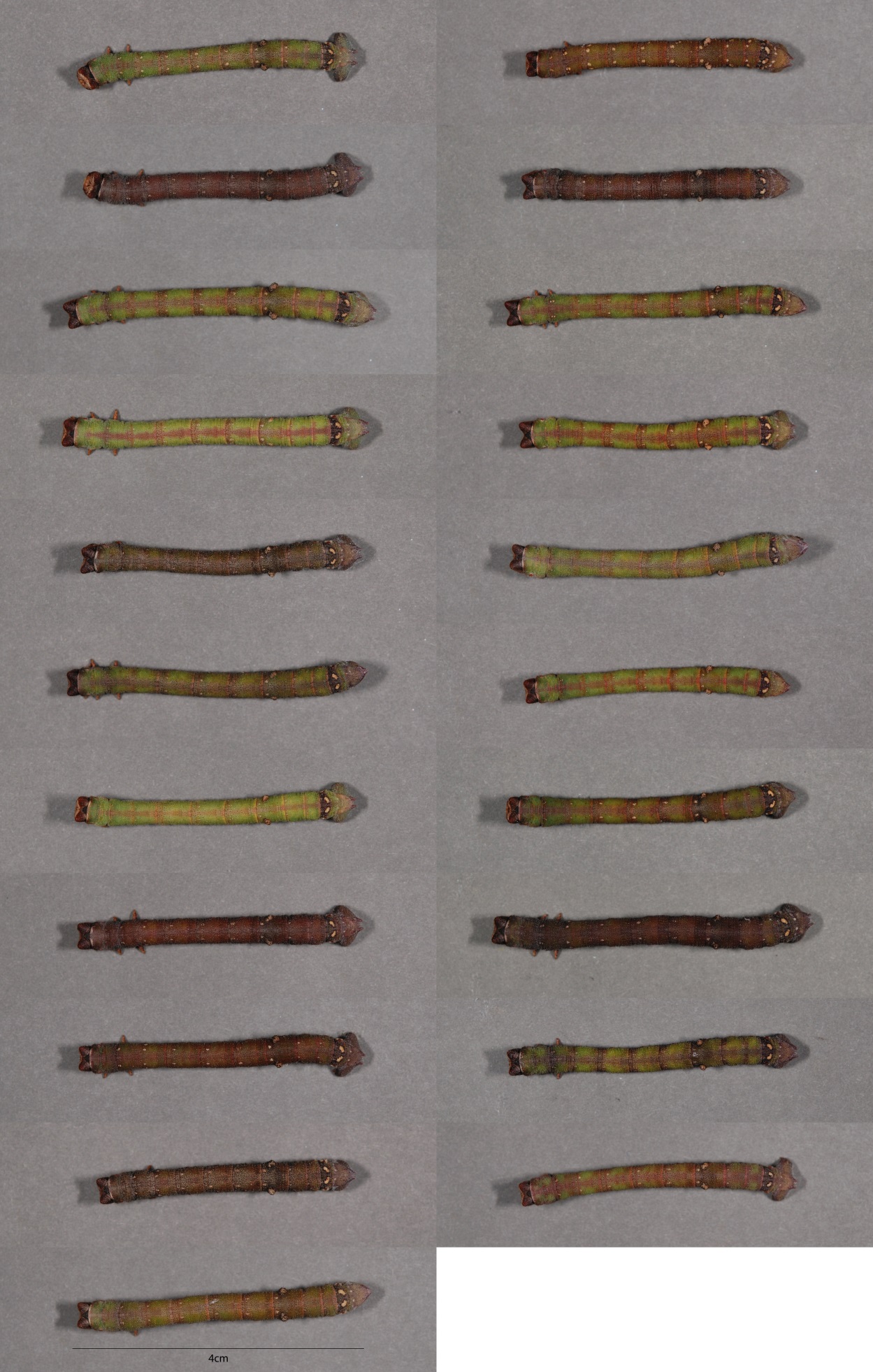


**Fig. S3B. Heterogeneous environment treatment**. Photographs of the final instar larvae under treatment 70G (70% Green, 30% brown dowel proportions).


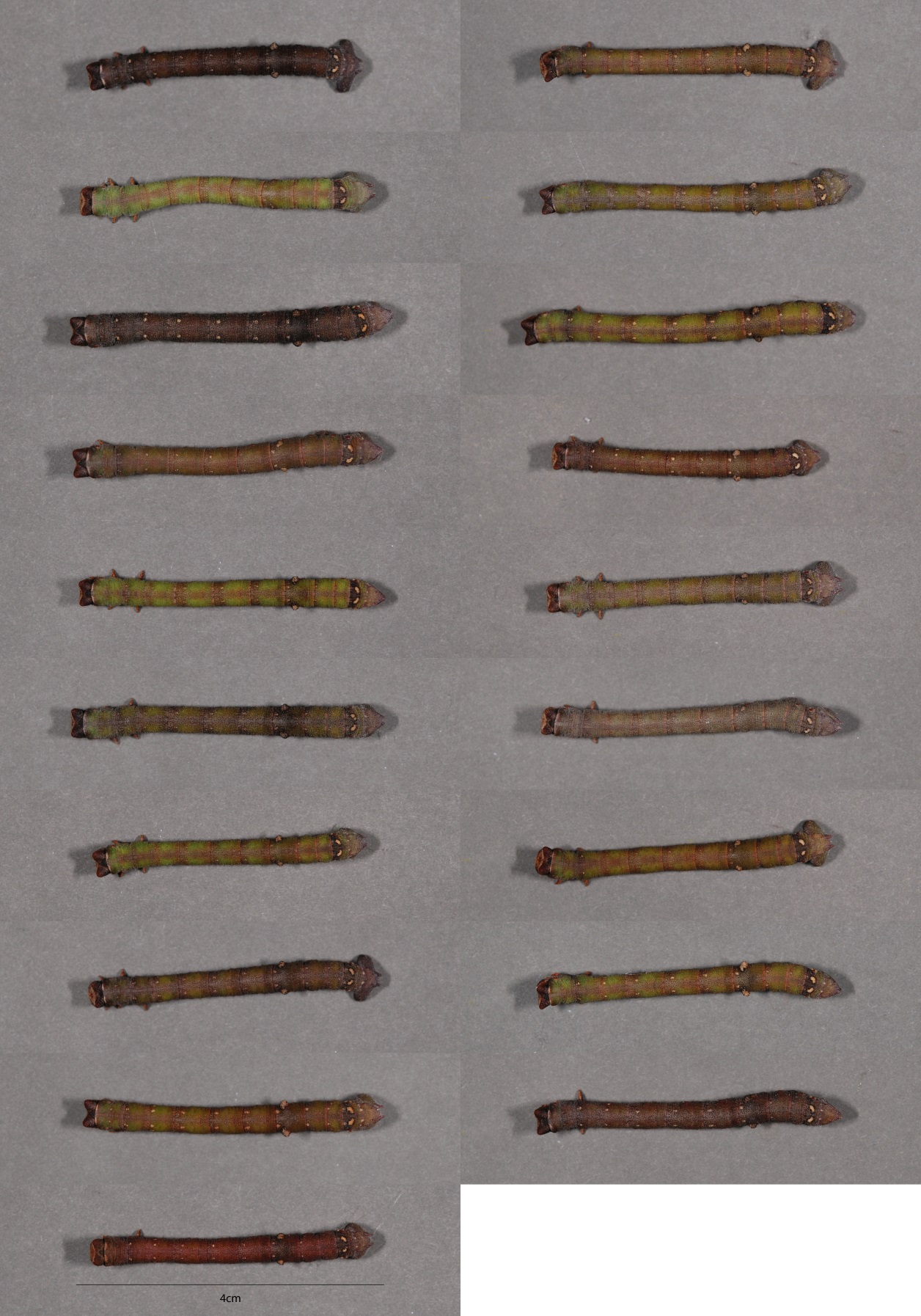


**Fig. S3C. Heterogeneous environment treatment**. Photographs of the final instar larvae under treatment 50G (50% Green, 50% brown dowel proportions).

**
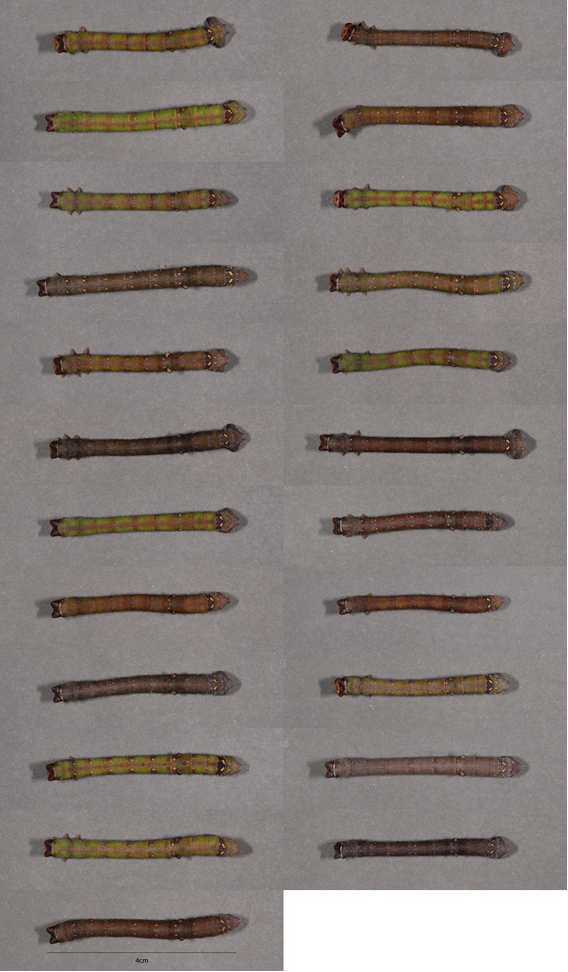
**

**Fig. S3D. Heterogeneous environment treatment**. Photographs of the final instar larvae under treatment 30G (30% Green, 70% brown dowel proportions).

**
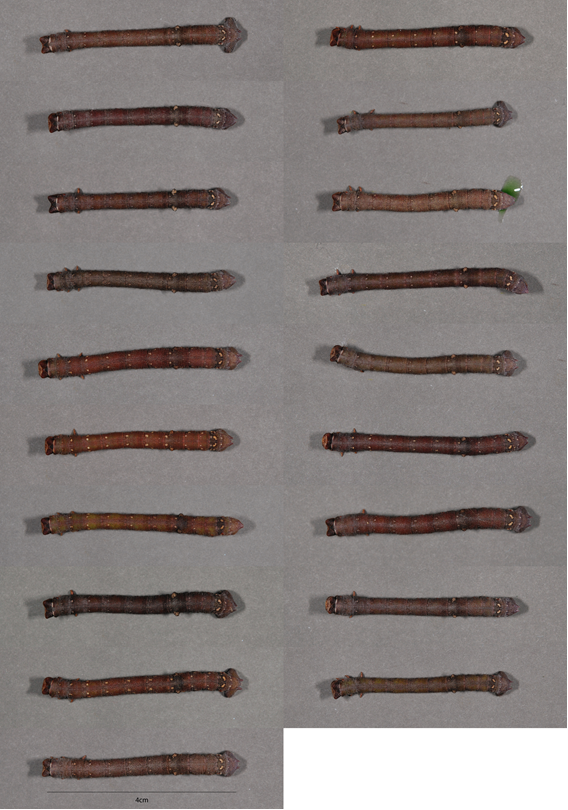
**

**Fig. S3E. Heterogeneous environment treatment**. Photographs of the final instar larvae under treatment 0G (0% Green, 100% brown dowel proportions).
