# Supplementary material for: Colour change of twig-mimicking peppered moth larvae is a continuous reaction norm that increases camouflage against avian predators
Source: PeerJ. 2017 Nov 14;5:e3999. doi: 10.7717/peerj.3999 (PMC5691783; doi:10.7717/peerj.3999)
Supplement: Table S1 [file peerj-05-3999-s005.docx]

**Table S1. Experimental design summary.**

| **Treatment name and label** | **Treatment** | **Dowel paint (Dulux)** | **Number replicate boxes** | **Adult melanic morph genotypes*** | **Sample size** |
| --- | --- | --- | --- | --- | --- |
| **Isoluminant dowels** | | | | | |
| IB | Isoluminant brown | Wild mushroom 1 | 5 | One family: *t/t*, *i/t* | 61 |
| IG | Isoluminant green | Indian ivy 2 | 5 |  | 76 |
| **Luminance gradient** | | | | | |
| Bl | Black | Night jewels 1 | 3 | Two families: *t/t* | 20 |
| BW1 | Dark grey | Night jewels 2 | 3 |  | 11 |
| BW2 | Mid grey | Grey steel 1 | 3 |  | 17 |
| BW3 | Light grey | Grey steel 2 | 3 |  | 9 |
| Wh | White | Chiffon white 4 | 3 |  | 20 |
| **Chroma and luminance gradient** | | | | | |
| Br | Brown | Espresso shot | 3 | Four families: *t/t*  One family: *t/t*, *c/t*, *c/c* | 42 |
| BG1 | Brown-green 1 more brown | 25:75 Indian ivy 3: Espresso shot | 3 |  | 67 |
| BG2 | Brown-green 2  50:50 brown green | 50:50 Indian ivy 3: Espresso shot | 3 |  | 67 |
| BG3 | Brown-green 3  More green | 75:25 Indian ivy 3: Espresso shot | 3 |  | 70 |
| Gr | Green | Indian ivy 3 | 3 |  | 34 |
| **Heterogeneous dowels** | | | | | |
| 0G | Heterogeneous dowels – 100% brown | Espresso shot | 1 | Two families: *t/t* | 19 |
| 30G | Heterogeneous dowels- 30% green, 70% brown | Indian ivy 3  Espresso shot | 1 |  | 23 |
| 50G | Heterogeneous dowels- 50% green, 50% brown | Indian ivy 3  Espresso shot | 1 |  | 19 |
| 70G | Heterogeneous dowels- 70% green, 30% brown | Indian ivy 3  Espresso shot | 1 |  | 21 |
| 100G | Heterogeneous dowels – 100% green | Indian ivy 3 | 1 |  | 22 |

* Expected genotypes of F_1_ larvae at the locus that determines the adult morph, based on known genotypes of the parents (alleles: *t* = *typica*; *i* = *insularia*; *c* = *carbonaria*).
